# Dengue infection during pregnancy and the occurrence of pathological neonatal outcome: a systematic review and meta-analysis

**DOI:** 10.12688/f1000research.158890.1

**Published:** 2024-12-16

**Authors:** Evelyn del Socorro Goicochea-Ríos, NELIDA MILLY OTINIANO, Lola del Carmen Rojas-Infantas, Víctor Raú Ocaña-Gutiérrez, Néstor Iván Gómez-Goicochea

**Affiliations:** 1 Universidad Cesar Vallejo, Trujillo, La Libertad, Peru; 2 Asociacion Civil Universidad Catolica De Trujillo Benedicto XVI, Trujillo, Peru

**Keywords:** Dengue, pregnancy, pregnancy complications, infant, newborn, pregnancy outcome, neonatal outcome.

## Abstract

**Background:**

Dengue infection during pregnancy increases the risk of maternal and neonatal complications; therefore, the objective of this research is to determine these outcomes and describe the clinical manifestations of the infection.

**Methods:**

A systematic review of studies published in PubMed, MEDLINE, LILACS, Web of Science, Scopus and thesis repositories published between 2013 and October 2023 was performed. DeCS and MeSH dengue and maternal-neonatal outcome were used. Thirteen studies were selected and the New Castle-Ottawa scale was used to assess their quality. Mantel-Haenszel hazard ratios were calculated to report the overall effect size using random-effects models. All analyses were performed in Rev Man 5.4.1

**Results:**

The 13 studies involved a population of 18,724 pregnant women, with cohorts ranging from 25 to 17,673 pregnant women. The most frequent outcomes in the pregnant women were cesarean section and postpartum hemorrhage, and in the newborns, preterm delivery and low birth weight. According to the New Castle-Ottawa scale, six studies were considered low risk and seven studies moderate risk. Dengue is a risk factor for postpartum hemorrhage (OR: 2.24), premature rupture of membranes (OR: 1.04) and cesarean section (OR: 1.13). It could not be concluded that dengue is a risk factor for the neonatal outcomes studied. The clinical picture of pregnant women with dengue was predominantly fever, abdominal pain, vomiting and nausea, anemia, dyspnea and myalgia.

**Conclusions:**

Pregnancy-related changes in the immune, cardiovascular and coagulation systems, among others, increase the probability of adverse maternal and neonatal outcomes in case of DENV infection, such as postpartum hemorrhage, premature rupture of membranes, cesarean section, low birth weight and preterm delivery. Pregnant women should be considered a population at risk and should be included in dengue control, diagnosis and treatment policies.

## Introduction

Dengue is a rapidly spreading tropical disease transmitted by mosquitoes of the genus *Aedes*, the most common being *Aedes aegypti.* The two most common forms of the disease affecting humans are dengue fever and severe dengue haemorrhagic fever. ^1^ Globally, the incidence of dengue is high in Southeast Asian countries (70% of the global burden of this disease), as well as in the Eastern Mediterranean and Western Pacific regions. ^1^ In the Americas, epidemic outbreaks are reported every 3 to 5 years, and the most affected countries include Central America, Mexico, Brazil, Venezuela, Peru, Colombia and Bolivia. ^2^ The number of cases of severe dengue has also increased markedly, especially in Brazil, Colombia, Peru, Bolivia and Mexico. ^3^

In the Americas, up to the epidemiological week 24-2023, 2,102,848 cases of dengue were reported, of which 39.3% were laboratory-confirmed and 0.15% were severe dengue cases, with a cumulative incidence rate of 214 cases/100,000 population. The highest number of dengue cases was observed in Brazil with 1,515,460 cases, followed by Peru with 169,504 cases and Bolivia with 133,452 cases. ^3^ Furthermore, dengue virus infection (DENV) is hyperendemic and should be addressed as a public health problem. ^4^ Affected countries are challenged to maintain the dengue case fatality rate < 0.05%. Therefore, timely diagnosis, early identification of warning signs, and appropriate treatment are important to avoid severe cases and deaths. ^3^

DENV infection is classified into dengue without alarm signs and dengue with alarm signs. In both cases, nausea and vomiting, rash, arthralgias, positive serological tests in population with other confirmed cases may occur. In the severe form, alarm signs include abdominal pain, mucosal bleeding, blood extravasation, general condition, hepatomegaly > 2 cm, and rapid decrease in platelets. ^5^ When dengue occurs in pregnant women, the risk to mother and child increases. Some studies have described the association between maternal dengue and pathologic neonatal outcomes as prematurity, low birth weight, ^6– 8^ postpartum haemorrhage, ^9– 11^ miscarriage, and increased rates of caesarean delivery. ^12^ Severe dengue infection can be difficult to differentiate from HELLP syndrome or gestational thrombocytopenia ^13^ and, as mentioned, can be complicated by postpartum hemorrhage. ^11^ The association of dengue infection with adverse fetal outcomes remains unclear, ^11, 13^ but prematurity, growth retardation and stillbirth can occur, especially in cases of severe maternal infection. According to other authors, the clinical picture of the newborn appears to be independent of the degree of severity of maternal dengue. ^6^

To date, research has been published reporting that DENV represents an ongoing threat to the population in endemic areas, particularly pregnant women who face more severe complications from this virus. These complications can severely affect pregnancy outcome, as it is associated with haemorrhage, fetal loss and preterm delivery. ^14^ It has also been documented that pregnant women are at higher risk of severe infection and of developing haemorrhagic fever/shock syndrome compared to non-pregnant women of reproductive age, with an OR of 3.38 and 95% CI. In the case of neonates, severe infection with sepsis-like symptoms and acute respiratory distress is documented. ^15^

Vertical transmission of DENV has been described in studies that analysed placental tissue from patients with confirmed infection during pregnancy, as well as immunohistochemical findings of monoclonal antibodies to dengue virus. ^10^ The risk of this transmission varies between 18.5% and 56.2% when infection is confirmed two weeks before delivery and up to two days postpartum. ^13, 16^ Although maternal and neonatal outcomes depend on the severity of DENV infection, the prevalence of infection and the socioeconomic costs associated with it require an updated review of the subject to promote preventive measures based on scientific evidence.

It has been reported that most of the reported cases occur in the economically active population, ^3^ and in Peru the most affected age groups are those aged 30-59 years and 18-29 years. In terms of sex, more than half of the cases correspond to the female sex ^2, 4^ which could mean that women of reproductive age acquire the infection during pregnancy.

There is considerable interest in identifying maternal-neonatal outcomes in patients with dengue infection during pregnancy, ^6, 7^ as a strategy for the timely detection of complications and to reduce the risk of death. However, there are few studies on the severity of the disease, clinical outcomes of pregnancy and delivery in pregnant women and their neonates.

Due to the physiological changes inherent to pregnancy, pregnant women are at greater risk of adverse effects and pathological neonatal outcomes when dengue infection occurs, depending on its severity. ^8, 9^ Therefore, the questions addressed in this review are: is dengue infection during pregnancy associated with pathological neonatal outcomes? And, what is the impact of dengue severity on gestational outcomes?

As mentioned, the prevalence of dengue is high in different countries in the world ^3^ and affects a large part of the population, including pregnant women and neonates. Therefore, the research questions formulated in this review investigate the relationship between DENV infection during pregnancy and pathological maternal-neonatal outcomes, as well as the clinical picture of dengue in pregnant women.

## Methods

**Figure 1.**
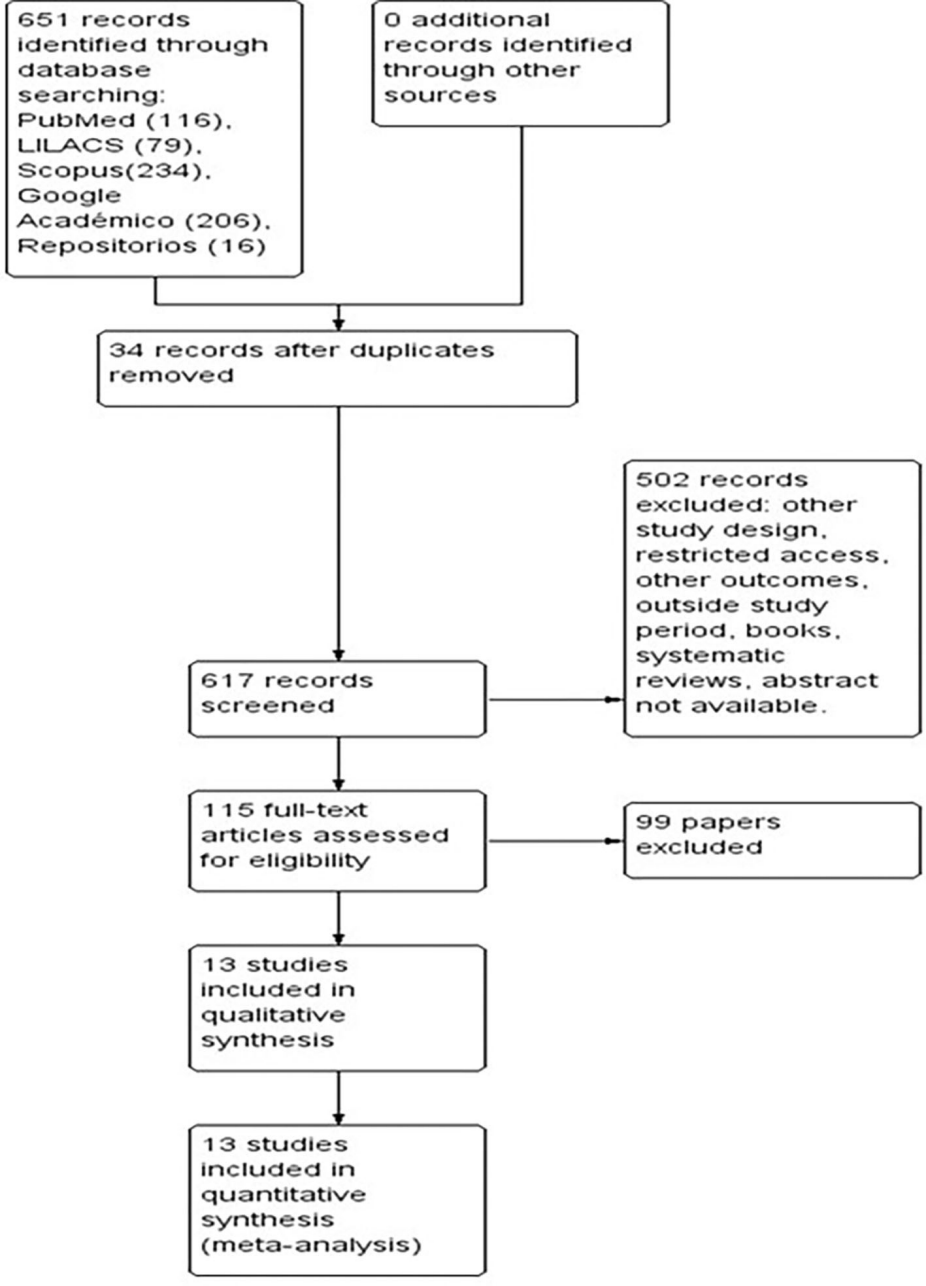
PRISMA flowchart describing the process of study selection.

This study was registered in the International Prospective Register of Systematic Reviews (PROSPERO, 2023) under number CRD42023453805 available from: https://www.crd.york.ac.uk/prospero/display_record.php? ID=CRD42023453805, and the recommendations of the Preferred Reporting Items for Systematic Reviews and Meta-Analyzes (PRISMA) were followed ^17^ ( Figure 1).

To identify the studies, an electronic search was carried out in the databases PubMed, Scopus, LILACS, WOS, Google Scholar, as well as in the WHO database, medical school repositories, conference proceedings and congress abstracts. No language or country restrictions were applied. Studies published between January 2013 and October 2023 were included. The search terms used were: pregnancy, pregnant, gestation, gravidez, dengue infection, neonatal outcome, recém nascido, dengue infection, infección por dengue, Febre da Dengue, Febre Quebra-Ossos, Infecção pelo Vírus da Dengue, Febre Hemorrágica Dengue.

The search strategy was adapted to each database. In PubMed, MeSH terms and Entry terms were included for title and abstract and the Boolean operators OR, AND, as well as truncation were used: Pregnancy [Mesh] OR Gestation [tiab] OR Pregnan*[tiab] AND Pregnancy [Mesh] OR Gestation*[tiab] OR Pregnan*[tiab]) AND Infant [Mesh] OR Newborn [tiab] OR Newborn Infant*[tiab] OR Neonate*[tiab] AND (Breakbone Fever [Mesh]) OR Classical Dengue Fever*[tiab] OR Break-Bone Fever [tiab] OR Fever, Break-Bone [tiab]. In LILACS the DECs were used: mh:(“Dengue/CN” OR “Dengue/CL” OR “Dengue/CN” OR “Dengue/EP” OR “Dengue/PP” OR “Dengue/TM”) AND (EMBARAZO/CN) AND (NEONAT*)

Duplicate citations were eliminated and all remaining articles containing the study variables in their titles and abstracts were reviewed.

### Criteria for eligibility

Articles that met the following inclusion criteria were included: case-control, cohort and cross-sectional full-text and open-access articles describing the clinical picture of dengue in pregnant women, as well as maternal and/or fetal/neonatal outcomes, such as cesarean section, abortions, preterm delivery, maternal hemorrhage, ICU admission and maternal mortality, stillbirths, low birth weight, small for gestational age and neonatal mortality.

Studies with a sample size of less than 10 were excluded, as well as systematic reviews, articles without statistical analysis, paid articles, reviews, letters to the editor, duplicate publications and clinical cases.

An Excel table was used to record the main data of each study and the reasons for inclusion or exclusion. The selection criteria for the studies included articles whose population was pregnant women with a diagnosis of dengue (regardless of the degree of severity) and the neonates of these pregnant women, published between January 2013 and October 2023. Articles that studied exposure to DENV infection were reviewed. Experimental interventions and pharmacological treatments were excluded.

### Types of outcome measures

The primary outcomes for the pregnant women were: a) clinical condition/comorbidity, b) complications: postpartum fever, postpartum hemorrhage, admission to the ICU, abortion and cesarean delivery. For neonates, primary outcomes included prematurity, low birth weight, small for gestational age, asphyxia, vertical transmission and admission to ICU. Secondary outcomes included maternal and neonatal mortality.

### Data extraction and analysis

*Selection of studies and quality assessment*

The Rayyan program ^18^ was used to collect all studies related to dengue during pregnancy and neonatal outcomes. All identified articles were independently reviewed by two reviewers for their inclusion in the study. Titles and abstracts of potentially relevant articles were reviewed in the first round, and the full text of those articles referring to dengue in pregnancy were retrieved and reviewed in detail to assess eligibility. Any disagreement between the two reviewers was resolved by a third reviewer until consensus was reached.

### Extraction and handling of the data

The data from each study were extracted independently by two authors, who performed a cross-check to eliminate errors. The included studies were organized in a standard template to record all relevant information, which included the surname of the first author, year of publication, country of origin, study design, place of patient recruitment, age, method of diagnosis, number of individuals included (case and control groups), sample size, number of pregnant women with dengue, dengue infection detection technique, gestational age, comorbidities (hypertensive disease of pregnancy, gestational diabetes) and clinical characteristics (fever, myalgia, malaise, dyspnea, diarrhea and joint pain). We also recorded outcomes related to type of delivery, premature rupture of membranes, postpartum fever, abortion, pre- and postpartum hemorrhage, and fetal/neonatal data: preterm delivery, low birth weight, fetal asphyxia, fetal/neonatal death, small for gestational age, and vertical transmission.

In the event that the requisite results for analysis were not forthcoming, the article was excluded in order to preclude the potential for reporting bias.

### Statistical analysis

The RevMan software (Review Manager 5.4.1) ^19^ was employed for the analysis of the collected data. The odds ratios with a 95% confidence interval were utilised, and the results were pooled using the Mantel-Haenszel model. The heterogeneity of the effect size was estimated using the I2 statistic, with the following ranges: I2 ≤ 25%, 25%–50%, and >75% (corresponding to low, moderate, and high, respectively).

In all cases, the probability values of less than 0.05 were deemed to be statistically significant. Furthermore, a leave-one-out sensitivity analysis was conducted to evaluate the influence of each individual study on the statistical outcomes. The risk of bias of the included studies was evaluated by two investigators using the Newcastle-Ottawa Scale (NOS) and its subscales. The assessment was conducted independently and considered three domains: selection of study groups, comparability of groups, and determination of exposure or outcome. The scoring system was applied to case-control and cohort studies, respectively. ^20^ The studies were classified according to the level of bias present, with categories of low (8-9 points), moderate (5-7 points), and high bias (0-4 points). The strength of evidence for all the outcomes analysed was evaluated using the GRADE tool, which is a recognised method for assessing the quality of evidence in systematic reviews. ^21^

### Strategy for data synthesis

A descriptive synthesis of the results was written, recording number of cases, percentages in tables for each maternal and neonatal outcome For maternal and neonatal outcomes, the OR, CI, and heterogeneity described in each included study were included. These data were analyzed using the version non-Cochrane of Revman 5.4.1. ^19, 22^ The data were tabulated in Excel. Plots from Rev Man ^19^ were also used to visually present the results of the individual studies and the syntheses. No subgroup analysis was performed

The grouped prevalence was calculated using the random-effects model. The results of the analysis of maternal-neonatal outcomes were presented as odds ratios with 95% confidence intervals. For some outcomes, high heterogeneity was found, so each study was analyzed independently.

## Results

### Characteristics of the included studies

The preliminary searches of the databases consulted identified a total of 651 studies. After eliminating 34 duplicate articles, 617 studies were screened, of which 502 were excluded because they corresponded to other study designs, had restricted access, dealt with other outcomes, were systematic reviews, among other reasons. We reviewed the full text of 115 articles that included the variables of interest for the study in the titles and abstracts. Of these, 99 articles were discarded because they did not meet the eligibility criteria. Finally, 13 articles that met the inclusion criteria were selected and their analysis is included in this review ( Figure 1).

Of the 13 included studies, 8 (62%) are retrospective cohort, 3 (23%) are prospective cohort and 2 (15%) are case-control. Of these studies, 5 were published in Latin America, 3 in Asia, 3 in India and 2 in Africa (Table 1 extended data). These studies reported data on a total of 18,724 DENV-infected pregnant women. Ninety-two percent of the studies focused on the clinical picture and maternal outcome, while 100% addressed neonatal outcome.

The most reported outcomes in the included studies were cesarean section and postpartum hemorrhage in pregnant women, as well as preterm delivery and low birth weight in neonates. Regarding the characteristics of the population, the sample size ranged from 25 to 17673 pregnant women. Dengue diagnosis was performed using the NS1 test (in 11 of the 13 studies) and IgM (in 12 of the 13 studies). According to the New Castle-Ottawa Scale score (NOS), six low-risk studies and seven moderate-risk studies were identified (Table 1 extended data).

**Figure 2.**
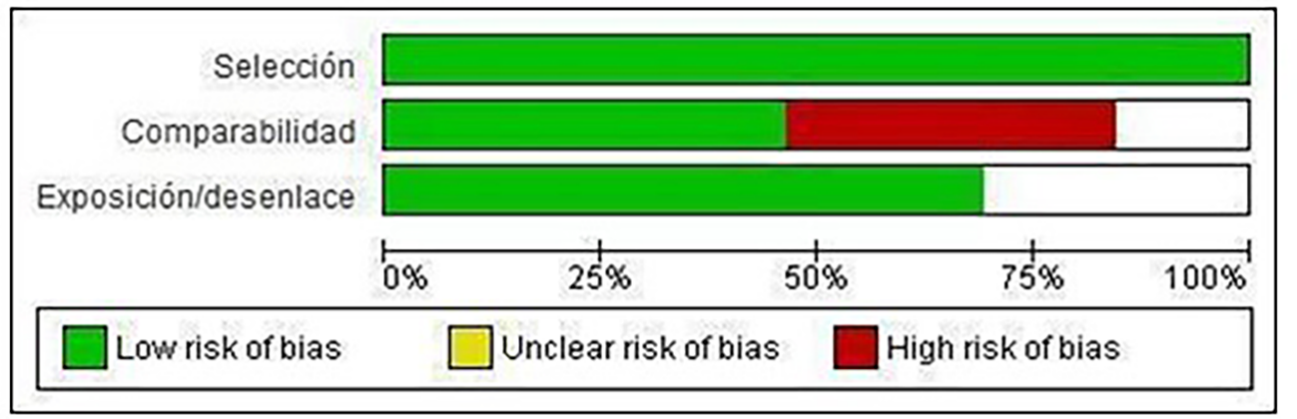
Risk of bias graph: review authors' judgments on each element of risk of bias, presented as percentages in all included studies.

Of the included studies, 100% meet the “selection” category established by NOS for both cohort studies and case-control studies. Forty-eight percent met the “comparability” category and 70% met the “exposure/disclosure” category ( Figure 2).

**Figure 3.**
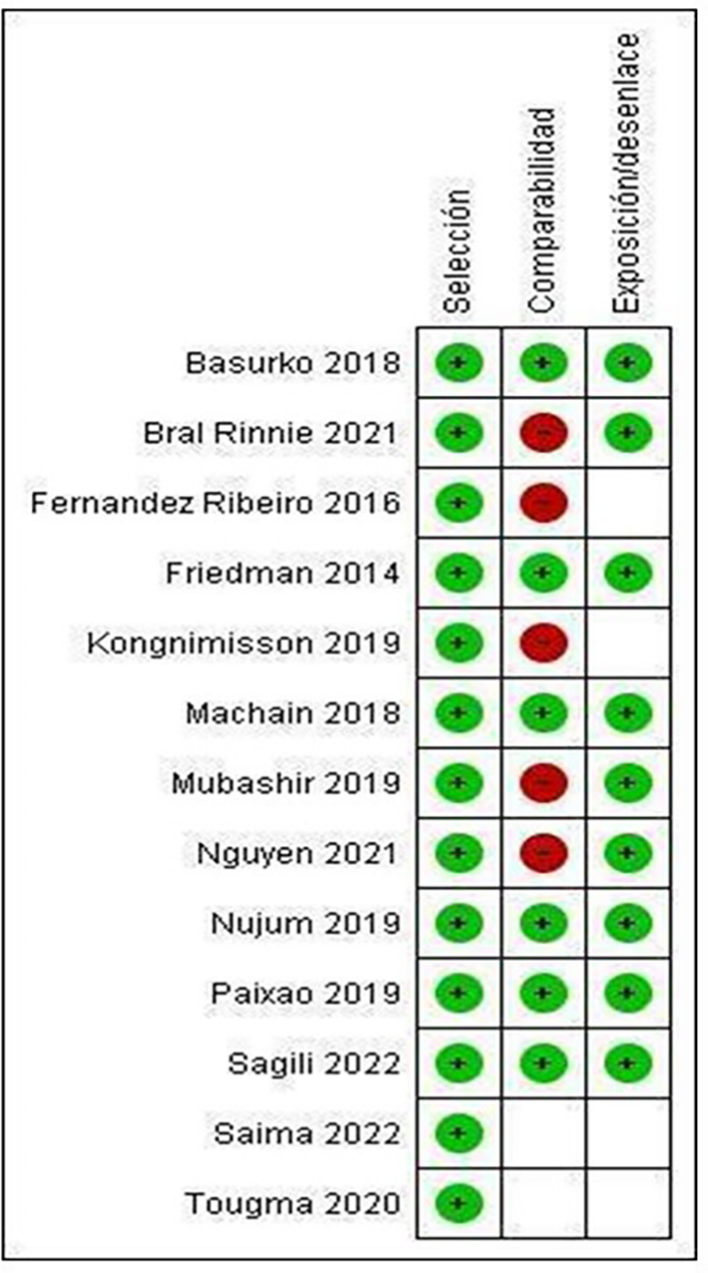
Summary of risk of bias: review authors' judgments on each element of risk of bias for each included study.

Only the studies by Basurko 15(2018), Friedman 25 (2014), Machain 23 (2018), Nujum 29 (2019), Paixao 14 (2019) and Sagili 31(2022) meet all the categories established by NOS, therefore presenting low risk of bias ( Figure 3).

Dengue was found to be a risk factor for postpartum haemorrhage (OR: 2.23) and caesarean section (OR: 1.39). In the case of neonatal outcomes, it cannot be concluded that dengue is a risk factor for low birth weight and preterm delivery due to OR <1 and high heterogeneity of the included studies (≥ 97%).

In the clinical picture of DENV infection, the following findings predominated: fever (7 studies); abdominal pain, vomiting and nausea and hypertensive disease of pregnancy (5 studies); anemia (4 studies), dyspnea, bleeding, myalgia and gestational diabetes (3 studies).

## Discussion

Of the 13 studies selected, it was found that more than 90% of pregnant women with dengue presented fever, ^12, 16, 23– 27^ followed by abdominal pain, ^15, 16, 23, 26, 28^ vomiting and nausea, ^12, 16, 23, 26, 28^ anemia, ^25, 27, 29, 30^ dyspnea ^16, 28, 26^ and myalgia ^12, 23, 26^ as the most frequent symptoms. Other publications also mention fever as the most frequent symptom, ^31, 32^ accompanied or not by headache, myalgias, arthralgias and other symptoms, ^33– 35^ although the clinical picture depends on the severity of dengue. ^31^ Among the most frequent comorbidities were hypertensive disease of pregnancy ^15, 27– 29, 36^ and gestational diabetes, ^15, 29, 36^ both pathologies referred to as frequent in patients with severe dengue. ^37^

Regarding maternal outcomes, 5 retrospective cohort studies report cesarean section, ^14, 16, 23, 25, 36^ as does a retrospective cross-sectional study ^26^ and the 2 case-control studies ^27, 30^ report postpartum hemorrhage, ^15, 26, 28, 30, 36^ miscarriages, ^28, 30, 36^ maternal deaths, ^16, 23, 26, 28, 36^ and premature rupture of membranes. ^15, 30^ To a lesser degree, ICU admission, ^16, 28^ thrombocytopenia, ^26^ oligohydramnios, ^28^ and antepartum hemorrhage are reported. ^15^ However, dengue alone was shown to be a risk factor for postpartum hemorrhage and cesarean section (OR >1) ( Table 2).

**Table 2.** Maternal and neonatal outcome in pregnant women with dengue in retrospective cohort studies.

Maternal outcome	Reference of the study	OR	CI (95%)	I2
Post-partum haemorrhage	27,28	2.23	0.24 – 20.38	0%
Cesarean section	14,16,23,25,28	1.39	0.38 – 2.41	61%
Neonatal outcome				
Low birth weight	10.14	0.48	0.40 – 0.56	100%
Pre-term delivery	10.28	0.02	0.01 – 0.03	97%

**Table 3.** Clinical picture of pregnant women with dengue fever.

Author/year/country	Type of study	Characteristics of the population	Clinical picture
Mujtaba Mubashir *et al* ^16^/ 2019/Pakistan	Retrospective cohort	N° pregnant women: 48 Age: 27.5 (25-33) Gestational age: 24 (19-35)	Fever: 42 (87.5%) Bleeding 9 (19%) Abdominal pain 7 (14.5%) Vomiting, nausea 6 (12.5) Dyspnea 4 (8%)
Machain-Williams Carlos *et al* ^23^/ 2018/Mexico	Retrospective cohort	N° pregnant women: 82 Age: 25.7 (16-36) Gestational age: 27.5 (16-39.4)	Fever: 82 (100%) Myalgia: 66 (80.5%) Vomiting and nausea: 40 (49) Abdominal pain: 15 (27.8) Diarrhea: 15 (18.3)
Nguyen Tuan M. *et al* ^24^/ 2021/Vietnam	Retrospective cohort	N° pregnant women: 32 Gestational age: 28.3 (25-31.4)	Fever: 4 (5%)
Fiedman Eleanor *et al*. ^25^/ 2014/Guayana Francesa	Retrospective cohort	N° pregnant women: 86 Maternal age: 26.6 Gestational age: 29.5 (7 to 40 weeks)	Anemia: 54 (62.79%) Fever: 22 (25.9%)
Fernandes Ribeiro Christiane *et al* ^12^/ 2016/Brasil	Retrospective cohort	N° pregnant women: 336 Gestational age: < 37 weeks: 14.6%. > 37 weeks: 85.4%	Fever: 17 (5.1%) Myalgia: 6 (2.4%) Vomiting, nausea: 1 (2.4%)
Tougma *et al*. ^36^/ 2020/Burkina Faso	Retrospective cohort	N° pregnant women: 95 Age: 27.1 (16-49 years old)	Hypertensive disease of pregnancy: 12 (2.83%) Gestational diabetes: 3 (0.71%)
Basurko Célia *et al* ^15^/ 2018/Guayana Francesa	Prospective cohort	N° pregnant women:73 Age: 28 (20-35) Gestational age: 28 weeks, 55%; between 14 and 28 weeks, 30%; before 14 weeks, 15%.	Bleeding: 8 (11%) Abdominal pain: 7 (10%) Hemoglobinopathy: 3 (4.1%) Preeclampsia: 3 (6.8%) Diabetes: 2 (2.7%) Autoimmune disease 2 (2.7%) Thromboembolism 1 (1.4%) Hypertension 1 (1.4%)
Brar Rinnie *et al* ^28^/ 2021/India	Prospective cohort	N° pregnant women: 44 Age: 24.5 (18-37 years) Gestational age: 31.89 weeks	Vomiting and nausea: 21 (47.50%) Acute respiratory distress syndrome: 8 (18.2%) Acute kidney injury: 8 (18.2%) Abdominal pain: 3 (6.8%) Hypertensive disease of pregnancy: 2 (5%)
Nujum Zinia T, *et al* ^29^/ 2019/India	Prospective cohort	N° pregnant women: 74 Age: 18-44 (mean 24 years)	Hypertensive disease of pregnancy: (n = 11) 14.90%. Gestational diabetes: 5 (6,.80%), Anemia 22 pregnant women (29.7%)
Kongnimissom Apoline Sondo *et al* ^26^/ 2019/Burkina Faso	Retrospective cross-sectional	N° pregnant women: 25 Age: 30 (18-45)	Fever: 23 (92%) Myalgia: 16 (64%) Vomiting/nausea: 14 (56%) Dyspnea: 9 (36%) Bleeding: 8 (32%) Abdominal pain: 4 (16%)
Naz Saima ^27^ *et al*/ 2022/Pakistan	Cases and controls	N° pregnant women: 65 Gestational age: 26.3 (20-31.7)	Arterial hypertension: 5 (7.7%) Anemia: 3 (4.6%) Fever: 1 (8.3%)
Sagili Haritha *et al* ^30^/ 2022/India	Cases and controls	N° pregnant women: 91 Age: 24.7 (20.9-28.5); Gestational age: 36 weeks	Anemia: 72 (79.1%)

Regarding newborn outcomes, fetal or neonatal death, ^15, 16, 25– 28, 30, 36^ is reported as the most frequent outcome, followed by pre-term delivery, ^12, 15, 27, 29, 30, 36^ low birth weight, ^12, 14, 27, 29, 30^ small for gestational age, ^14, 15, 30^ admissions to the ICU, ^16, 27, 30^ hepatomegaly, neonatal asphyxia, neonatal infection, and vertical transmission. ^24^ However, it is inconclusive that dengue is a risk factor for low birth weight, pre-term delivery, and small for gestational age, (OR < 1), in addition to the high heterogeneity of the included studies (I ^2^ >97%).

Other research reports that no relationship was found between dengue infection and preterm delivery. ^38^ In other studies, the risk of pre-term delivery was higher in dengue seropositive than seronegative groups. in all trimesters of pregnancy (OR: 1-26, 95% CI 1-06-1-49, p=0-006). ^39^ Spontaneous abortion linked to dengue infection could not be analyzed because the studies corresponded to different designs. On the other hand, there was no significant association between maternal dengue infection and fetal death in two cohort studies involving a total of 572 participants. (grouped RR 3.42, 95% CI 0.76-15.49, I2 = 54.8%). ^38^ This contrasts with another investigation in which dengue infection was found to be associated with high maternal and perinatal mortality. ^40^

Maternal dengue was shown to be associated with a slightly increased risk of preterm birth and low birth weight, although the CI was borderline. This review did not identify a notable correlation between dengue infection during pregnancy and low birthweight (LBW). However, the findings indicated that dengue haemorrhagic fever during pregnancy was associated with a twofold increased risk of preterm birth (OR 2.4, 95% CI 1.3 to 4.4) and an elevated likelihood of LBW (OR 2.1, 95% CI 1.1 to 4.0). The study found no correlation between maternal dengue and low weight for gestational age. ^41^

During pregnancy, there are changes in the immune, cardiovascular and coagulation systems, as well as liver enzymes and febrile response. The white blood cell count is usually elevated, with a leftward shift, and the platelet count is usually low. Therefore, the changes produced by dengue may go unnoticed, which justifies the need to clarify this diagnosis when a pregnant woman presents with fever. ^42, 43^ Some pathophysiological mechanisms have been proposed for dengue infection For example, the up-regulation of proinflammatory cytokines (interleukin 6 (IL-6), interleukin 8 (IL-8) and tumour necrosis factor alpha (TNF-α)) may result in alterations to normal gestational physiology. It is important to be aware that thrombocytopenia, plasma leakage or bleeding tendency may affect placental circulation and cause fetal complications. In severe dengue infection, there is a risk of endothelial damage and increased vascular permeability, which could allow DENV to cross the placental barrier. ^42^

During the normal gestation period, there is an overall increase in innate immune cells and effector mechanisms, as well as an increase in complement activity. Furthermore, there is a notable rise in the number of circulating phagocytes and plasmacytoid dendritic cells (DCs) producing type I interferon (TNF-1). It has been demonstrated that the innate pathways responsible for defending against retroviruses are specifically enhanced during pregnancy. To illustrate, IFN-induced activation of signal transducer and activator of transcription 1, a vital response to viral infection that is increased in natural killer cells, monocytes and myeloid dendritic cells (DCs) during gestation. ^43^ The innate antiviral response to DENV is regulated by a variety of immune cells and inflammatory mediators. When in balance, these allow for effective viral clearance, resulting in either asymptomatic infections or mild clinical manifestations. An imbalance between the components of the immune response can lead to increased immune activity and disease severity. ^44^

It is thought that placental tropism, changes in innate immunity and vascular permeability during normal pregnancy may contribute to an increased susceptibility to severe DENV infection in pregnant women. in combination with excessive or insufficient maternal innate immune system activity. The effects of DENV infection include a reduction in the number of megakaryocytes, which can lead to thrombocytopenia. Additionally, there is an increase in the expression of interferon-induced transmembrane protein 3 in platelets, resulting in the release of type I IFN. This could contribute to the increased incidence of haemorrhagic complications during pregnancy. There is evidence that RNA virus infections are connected with an elevated risk of miscarriage, haemorrhage and foetal death. ^43^

It has been described that, at the maternal-fetal interface, NK cells, macrophages, DCs and T cells can modify their function and secretome during viral infections. These changes can lead to complications such as premature birth, congenital malformations, miscarriage or fetal death, and hematoplacental transmission. ^4, 43^

Complications vary according to the time of gestation. In the first trimester there is an increased risk of pre-eclampsia and eclampsia, especially in cases of severe dengue. Plasma leakage is the result of inflammatory cytokines, DENV non-structural protein-1 (NS1) and inflammatory lipid mediators have been identified as factors that increase capillary permeability. It is possible that maternal compensatory mechanisms for pathologic plasma leakage and vasodilatation are a contributing factor in the development of preeclampsia. In addition, the decrease in pulse pressure to 25 mm Hg or less, along with shock and dengue fever, increases capillary permeability. This further increases the risk of acute pulmonary oedema for infected mothers. Decreased plasma volume may also contribute to bleeding in dengue, although there is no specific evidence for the third trimester. ^41, 45^

The role of hormones in dengue complications in pregnant women is not completely clear. In the first trimester, leukopenia may not be evident. Due to the physiological effects of haemodilution and haemoconcentration with plasma leakage, this may be masked in cases of dengue. Accordingly, an elevated hematocrit may be indicative of maternal shock. In the second trimester thrombocytopenia may occur, and a decline in platelet levels <80 × 100/L may suggest plasma leakage. Decreased oncotic pressure and associated pulmonary resistance may lead to acute pulmonary edema. In the third trimester, the risk of vertical transmission of the virus is greatest, ^42, 46^ and during the postpartum period, there is evidence of transmission through breastfeeding. ^42, 45^

In this review, we found that most articles report performing cesarean section in patients with dengue (10/13). On this point, the studies favor conservative treatment, unless there is some obstetric reason to intervene since a cesarean section may be precipitated in an unstable patient with anesthetic risks (such as spinal anesthesia), bleeding tendency or risk of hemorrhage during the intervention. Before term, there appears to be little indication for induction of labor or other obstetric interventions. ^47^

Pregnant women represent a high-risk group for serious complications associated with dengue infection, and it is estimated that maternal mortality is three times higher in pregnant women than in non-pregnant women with dengue. Thrombocytopenia and elevated liver enzymes c are associated with certain disorders of pregnancy, such as preeclampsia, HELLP syndrome (hemolysis, elevated liver enzymes and low platelets) or gestational thrombocytopenia, so it is crucial to make an accurate diagnosis by confirmatory serological tests for dengue.

Several studies report thrombocytopenia as one of the most common clinical findings ^48– 50^ in pregnant women with dengue fever, affecting between 40% and 70% of these patients. Severe hypovolemic shock, caused by severe hemorrhage and preeclampsia, could be favored by the state of plasma leakage. The severity of the disease could significantly influence the occurrence of maternal complications. ^10^ On the other hand, it has been observed that patients with dengue during the first trimester suffered spontaneous abortions, possibly due to inflammatory changes in the placenta. ^45^

The effects of dengue on the fetus or newborn appear to be variable. Apparently, fetal damage is less in early pregnancy, when there is sufficient time for maternal antibodies to transfer to the newborn. In contrast, if maternal infection occurs late in gestation, the risk of pathological neonatal outcomes increases. ^47^ Placental endothelial damage and increased placental vascular permeability influence adverse fetal outcomes. ^51^ In addition, vertical transmission from mother to foetus can cause developmental abnormalities, growth retardation, preterm birth and stillbirth. At the same time, the mother is predisposed to increased morbidity and death. ^52, 53^

As mentioned, DENV induces pathological changes related to increased production of proinflammatory cytokines ^42, 54^ which can affect the uterus by stimulating the production of proteins that promote uterine contractions, potentially leading to preterm delivery. ^55^ In addition, among these proinflammatory cytokines is TNF-α, which is related to bleeding, favoring thrombocytopenia and endothelial dysfunction ^56, 57^; in addition, antibodies are produced that cross-react with plasma proteins, platelets and the endothelium, which also leads to hemorrhagic symptoms. ^56^

Therefore, the thrombocytopenia, plasma leakage or bleeding tendency inherent to the disease could cause damage to the placental circulation, with adverse effects on the fetus, including the risk of stillbirth. ^55^ In addition, endothelial damage and increased vascular permeability make it easier for DENV to cross the placental barrier. ^42^

The association between dengue and pre-term delivery ^10, 54^ as well as low birth weight has been documented in relation to the severity of the disease. ^55^ It is frequently observed that both outcomes occur in pregnant women affected by dengue, which can have serious effects on the health and development of the child. Furthermore, the occurrence of stillbirths, especially in women with severe dengue, lends support to this hypothesis. ^54^ Likewise, the relationship between dengue and intrauterine growth retardation is also reported. A meta-analysis including six cohort studies and two case-control studies found an association with pre-term delivery (OR 1.71 (95% CI 1.06-2.76), between miscarriages in pregnancies and dengue infection compared to those without infection (OR 3.51; 95% CI 1.15-10.77) However, no significant association was found with low birth weight. ^10^

It is important to note that some studies showed an increased rate of prematurity and fetal/neonatal death, ^10, 54^ while others have found no significant fetal effects, such as preterm delivery, fetal death, low birth weight ^42^ or other maternal complications, such as miscarriage and maternal hemorrhage. This suggests the need for further research to clarify these discrepancies. ^47^ Available studies indicate that adverse maternal and perinatal outcomes are more frequent in women with severe forms of dengue, especially when infection occurs near the end of gestation. ^49, 58, 59^

It is possible that placental ischaemia could have an impact on foetal growth. A study conducted in Brazil indicates that capillary leak syndrome and capillary permeability may result in inadequate vascular supply to the foetus. This could potentially lead to the development of hypoxic lesions, such as involvement of the trophoblastic epithelium or chorangiosis, as well as inflammatory lesions affecting the decidua or choriodecidua. Hypoxia has been shown to be sufficient to reduce fetal growth rates. ^45^

Weaknesses of this research include the fact that more than 50% of the selected studies have a moderate risk according to the New Castle-Ottawa Scale (NOS). In addition, the studies on neonatal outcomes have varied research designs and high heterogeneity, and 4 of the 13 studies included small samples (less than 50 cases). We also found no evidence on the virus serotype affecting pregnant women and its influence on maternal and neonatal outcomes. The existence of asymptomatic cases and the difficulties associated with accurate diagnosis of acute dengue make it challenging to define the risks associated with DENV infection during pregnancy. This is due to the fact that dengue IgM can cross-react with other flaviviruses, and co-infections are common. ^43^

We consider that these results show risk factors for pathological outcome in DENV infections during pregnancy, both for the pregnant woman and for the newborn. It is essential to take these factors into consideration in the application of preventive measures and in monitoring the evolution of the disease. It also reinforces the need to hospitalize any pregnant woman with dengue symptoms and to manage the newborn of a mother with dengue as a high-risk case. Clinical, laboratory and ultrasound monitoring of pregnant women with dengue is crucial to detect possible adverse outcomes and improve clinical practice.

Given that in this review the studies do not provide evidence on the influence of a history of previous dengue infection on maternal-neonatal outcomes or on the impact of the serotype of the virus affecting pregnant women, it is recommended that homogeneous cohort or multicenter studies be conducted to generate better evidence regarding the role of dengue as a risk factor for pathological outcomes.

## Conclusions

The susceptibility generated by the physiological changes of pregnancy increases the probability of adverse outcomes in maternal, fetal and neonatal morbidity and mortality. Therefore, given the high prevalence of this disease worldwide, it is of utmost importance to establish more efficient guidelines for the detection, diagnosis and treatment of DENV infections, as well as to implement personalized care programs for the obstetric population. It would also be important to include in dengue control policies the recommendation to hospitalize all pregnant women with dengue fever for monitoring until the disease has been eliminated.

However, it remains to be established which are the initial and most determinant elements in the immunopathogenesis of dengue during pregnancy. A better understanding of these events will allow the development of effective therapeutic measures to prevent severe forms of the disease in pregnant women. It is crucial to consider the temporal/spatial component in the evolution of clinical cases in future studies, as this will improve our understanding of the relevance of each effector of the immune response in DENV infections and immunopathogenesis during pregnancy.

## Ethics and consent

Ethical approval and consent were not required.

## Authors’ contributions

**Evelyn Goicochea-Ríos:** Conceptualization, formal analysis, methodology, data collection, investigation, visualisation, writing – original draft preparation, writing – review & editing, funding acquisition.

**Nélida Milly Otiniano:** Methodology, data collection, formal analysis, writing – original draft preparation, writing – review & editing.

**Víctor Ocaña Gutiérrez:** Data collection, formal analysis, methodology, writing – original draft preparation, writing – review & editing.

**Lola Rojas Infantas**: Data collection, formal analysis, methodology, writing – review & editing.

**Néstor Gómez Goicochea:** Data collection, formal analysis, methodology, writing review & editing.

## Data Availability

No data are associated with this article. Zenodo: Dengue infection during pregnancy and the occurrence of pathological neonatal outcome. A systematic review and meta-analysis, 10.5281/zenodo.14278897. ^60^ This project contain the following underlying data:
1. Flow chart PRISMA Dengue in pregnancy.jpg 2. Database with results Dengue in pregnancy.xlsx 3.
PRISMA_2020_checklist RS dengue in pregnancy.docx 4. RS DENGUE IN PREGNACY RESULTS AND NOS (1) (2).xlsx 5.
Table 1 Dengue infection during pregnancy and the ocurrence of pathological neonatal outcome.docx Data are available under the terms of the Creative Commons Attribution 4.0 International license (CC-BY 4.0).
