# Supplementary material for: Dengue infection during pregnancy and the occurrence of pathological neonatal outcome: a systematic review and meta-analysis
Source: F1000Res. 2025 Aug 26;13:1523. Originally published 2024 Dec 16. [Version 3] doi: 10.12688/f1000research.158890.3 (PMC12338469; doi:10.12688/f1000research.158890.3)
Supplement: Peer review history

# Reviewer report: Reviewer response for version 2

**DOI:** 10.5256/f1000research.177785.r392301

**Published:** 2025-08-07

**Authors:** Jesus Trevino

**Affiliations:** 1 Universidad Autónoma de Nuevo León, Nuevo León, Mexico

**Overall Assessment**

 This article presents a timely and well-executed systematic review and meta-analysis that explores the association between dengue virus (DENV) infection during pregnancy and adverse maternal and neonatal outcomes. The authors adopt a rigorous methodological framework following PRISMA guidelines, register the protocol in PROSPERO, and provide a comprehensive literature synthesis. The paper makes a valuable contribution to maternal-fetal health literature, particularly in dengue-endemic regions.

**Strengths**

1. **Relevance and Originality**
  The study addresses a crucial knowledge gap regarding the effects of DENV infection in pregnant women and their offspring. The focus on vertical transmission, immunopathogenesis, and trimester-specific risks contributes to an underexplored but highly relevant field.
2. **Robust Methodology**
  The review adheres to PRISMA standards, includes a registered protocol, and applies systematic database searches without language or country restrictions. The use of the Newcastle-Ottawa Scale (NOS) and GRADE system adds methodological robustness.
3. **Detailed Clinical Insights**
  The discussion integrates clinical findings (e.g., fever, hemorrhage, ICU admission, fetal death), immunological mechanisms (e.g., cytokine cascades, placental permeability), and pathophysiological context across trimesters. This comprehensive synthesis is useful for clinicians and public health professionals.
4. **Transparency and Data Sharing**
  The inclusion of extended data (e.g., PRISMA diagram, data tables) via Zenodo reflects good research transparency and adherence to open science principles.

**Areas for Improvement**

1. **High Heterogeneity and Interpretation of Results**
  Many outcome measures exhibit considerable heterogeneity (e.g., I² > 90% for neonatal outcomes), which limits the conclusiveness of the pooled results. While the authors acknowledge this, consider adding a line to suggest that more emphasis is needed on interpreting these findings cautiously, perhaps by suggesting future subgroup analyses by region, trimester, or serotype.
2. **Underdeveloped Discussion on Viral Serotypes**
  The paper notes that no study reports serotype-specific effects; however, it is worth mentioning in the text that this limitation could be addressed with a more thorough call for virological surveillance data in maternal cohorts, as serotype may influence disease severity and vertical transmission risk.
3. **Lack of Subgroup or Meta-Regression Analyses**
  Given the observed heterogeneity, subgroup analyses (e.g., by trimester or geographic location) or meta-regression could clarify effect modifiers. Mention in the text how the absence of such analyses limits the granularity of interpretation.
4. **Language and Structure**
  While generally well-written, the manuscript would benefit from more concise and coherent paragraph transitions, particularly in the discussion section. Some redundancy (e.g., repeated references to plasma leakage and cytokine storms) could be streamlined.

**Specific Comments**

- **Introduction**: Clearly outlines the scope and relevance, but it is helpful to distinguish between general and pregnancy-specific dengue risks earlier in the narrative.
- **Methods**: Comprehensive and replicable. However, consider inserting a line on the bias assessment more clearly in the main text.
- **Results**: Well-organized with supportive figures and tables. Table 2 and the bias assessment visuals are beneficial. Mention how an exploration of potential publication bias (e.g., funnel plot or Egger's test) would strengthen the statistical review.
- **Discussion**: Strong synthesis of findings and biological mechanisms. Refer to how future directions could be made more actionable, such as advocating for standardized dengue surveillance protocols during pregnancy in endemic areas.
- **Conclusion**: Thoughtful and supported by the evidence. Mention how conclusions could be more assertive in recommending policy and clinical practice changes (e.g., integrating dengue screening in antenatal care).

**Recommendation**

 The article meets high standards for a systematic review in public health and infectious disease. Minor revisions related to clarity, discussion of limitations, and structure would further enhance its impact. The review is highly relevant to clinicians, researchers, and policymakers concerned with maternal health in areas endemic to dengue.

Are the rationale for, and objectives of, the Systematic Review clearly stated?

Yes

Is the statistical analysis and its interpretation appropriate?

Yes

If this is a Living Systematic Review, is the ‘living’ method appropriate and is the search schedule clearly defined and justified? (‘Living Systematic Review’ or a variation of this term should be included in the title.)

Yes

Are sufficient details of the methods and analysis provided to allow replication by others?

Yes

Are the conclusions drawn adequately supported by the results presented in the review?

Yes

Reviewer Expertise:

Spatial statistics and the spatial distribution of population and its problems.

I confirm that I have read this submission and believe that I have an appropriate level of expertise to confirm that it is of an acceptable scientific standard.

# Reviewer report: Reviewer response for version 2

**DOI:** 10.5256/f1000research.177785.r365511

**Published:** 2025-02-19

**Authors:** Phuc Nhon Nguyen

**Affiliations:** 1 Tu Du Hospital, Ho Chi Minh City, Vietnam

Thank you for revision. The paper is more well-improved.

Are the rationale for, and objectives of, the Systematic Review clearly stated?

Partly

Is the statistical analysis and its interpretation appropriate?

Partly

If this is a Living Systematic Review, is the ‘living’ method appropriate and is the search schedule clearly defined and justified? (‘Living Systematic Review’ or a variation of this term should be included in the title.)

Not applicable

Are sufficient details of the methods and analysis provided to allow replication by others?

Partly

Are the conclusions drawn adequately supported by the results presented in the review?

Partly

Reviewer Expertise:

Obstetrician at the Department of Pathologic Pregnancy

I confirm that I have read this submission and believe that I have an appropriate level of expertise to confirm that it is of an acceptable scientific standard.

# Reviewer report: Reviewer response for version 1

**DOI:** 10.5256/f1000research.174540.r357117

**Published:** 2025-01-20

**Authors:** Phuc Nhon Nguyen

**Affiliations:** 1 Tu Du Hospital, Ho Chi Minh City, Vietnam

Thank you for your work. I have some concerns:

 - In abstract, the OR should be provided with 95%CI.

 - Serotypes of DENV should be provided in introduction.

 - No language or country restrictions were applied. The search terms used were: pregnancy, pregnant, gestation, gravidez, dengue infection, neonatal outcome, recém nascido, dengue infection, infección por dengue, Febre da Dengue, Febre Quebra-Ossos, Infecção pelo Vírus da Dengue, Febre Hemorrágica Dengue.

 I concern if the same publication in different language. English search should be only applied.

 - Results should be expanded with more findings.

 - Table 1 is missing.

 - Table 2 should be presented as Forest plot.

 - Perinatal transmission should be mentioned in discussion. Pham TH, et al., 2023 (Ref 1)

 - Similar study had been reported. Rathore SS, et al., 2022 (Ref 2)

 - Recent systematic study included 20 studies. The authors could discuss on this point “While maternal dengue infection was associated with a higher prevalence of preterm birth and LBW, the associations were not statistically significant. Significant associations were observed for stillbirth in specific studies.”  Shabil M, et al., 2024 (Ref 3)

 - Conclusion is very common. Please focus the main findings before recommendation. For ex: “Pregnancy-related changes in the immune, cardiovascular and coagulation systems, among others, increase the probability of adverse maternal and neonatal outcomes…” or “The susceptibility generated by the physiological changes of pregnancy increases the probability of adverse outcomes in maternal, fetal and neonatal morbidity and mortality”

 - Full-word should be provided by the abbreviation word when it first appears in each part. For example: DeCS, HELLP syndrome

 - English editing is required. For example: This sentence should be reconstructed. “Given that in this review the studies do not provide evidence on the influence of a history of previous dengue infection on maternal-neonatal outcomes or on the impact of the serotype of the virus affecting pregnant women, it is recommended that homogeneous cohort or multicenter studies be conducted to generate better evidence regarding the role of dengue as a risk factor for pathological outcomes.”

Are the rationale for, and objectives of, the Systematic Review clearly stated?

Partly

Is the statistical analysis and its interpretation appropriate?

Partly

If this is a Living Systematic Review, is the ‘living’ method appropriate and is the search schedule clearly defined and justified? (‘Living Systematic Review’ or a variation of this term should be included in the title.)

Not applicable

Are sufficient details of the methods and analysis provided to allow replication by others?

Partly

Are the conclusions drawn adequately supported by the results presented in the review?

Partly

Reviewer Expertise:

Obstetrician at the Department of Pathologic Pregnancy

I confirm that I have read this submission and believe that I have an appropriate level of expertise to state that I do not consider it to be of an acceptable scientific standard, for reasons outlined above.

## References

[1] : Perinatal Transmission of Dengue Infection among Dengue Hemorrhagic Fever Outbreaks in Southern Vietnam: The First Case Managed at Tu Du Hospital and Review of Literature. *Am J Trop Med Hyg* .2023;108(1) : 10.4269/ajtmh.22-0572 155-160 10.4269/ajtmh.22-0572 36375466 PMC9833081

[2] : Maternal and foetal-neonatal outcomes of dengue virus infection during pregnancy. *Trop Med Int Health* .2022;27(7) : 10.1111/tmi.13783 619-629 10.1111/tmi.13783 35689528

[3] : Dengue Infection During Pregnancy and Adverse Birth Outcomes: A Systematic Review and Meta-Analysis. *Rev Med Virol* .2024;34(5) : 10.1002/rmv.2582 e2582 10.1002/rmv.2582 39245582

# Response

**Published:** 2025-01-31

**Authors:** NELIDA MILLY OTINIANO

Responses to observations:

**O: **In abstract, the OR should be provided with 95%CI.

**R: **In the summary, 95% CIs were added.

**O:** Serotypes of DENV should be provided in introduction

**R:**DENV serotypes were added in the introduction.

**O:**  No language or country restrictions were applied. The search terms used were: pregnancy, pregnant, gestation, gravidez, dengue infection, neonatal outcome, recém nascido, dengue infection, infección por dengue, Febre da Dengue, Febre Quebra-Ossos, Infecção pelo Vírus da Dengue, Febre Hemorrágica Dengue. I concern if the same publication in different language. English search should be only applied.

**R:** The search for articles was performed without country restriction and considering terms in English, Portuguese and Spanish. Duplicate articles were excluded. The article was written in English only.

**O: **Results should be expanded with more findings.

**R: **It was done, We are aggregating the results of small for gestational age and Table 1.

**O:** Table 1 is missing.

**R: **Table 1 was added. Due to the editor's indications, this table was placed in the repository because of its size.

**O: **Table 2 should be presented as Forest plot.

**R:** Table 2 is a summary of each of the maternal and neonatal outcomes. We have not used a forest plot because for one outcome, the included studies could have different designs (retrospective cohort, prospective cohort or case-control) and cannot be mixed for analysis.

**O: **Perinatal transmission should be mentioned in discussion. Pham TH, et al., 2023 (Ref 1)

**R:** It was added in discussion (ref 55)

**O: **Similar study had been reported. Rathore SS, et al., 2022 (Ref 2)

**R:** we quoted Rathore in the discussion (ref 43)

**O:** Recent systematic study included 20 studies. The authors could discuss on this point “While maternal dengue infection was associated with a higher prevalence of preterm birth and LBW, the associations were not statistically significant. Significant associations were observed for stillbirth in specific studies.”  Shabil M, et al., 2024 (Ref 3)

**R:** Shabil was added in discussion (ref. 42)

**O: **Full-word should be provided by the abbreviation word when it first appears in each part. For example: DeCS, HELLP syndrome

**R:** HELLP Syndrome was corrected

**O:** English editing is required. For example: This sentence should be reconstructed. “Given that in this review the studies do not provide evidence on the influence of a history of previous dengue infection on maternal-neonatal outcomes or on the impact of the serotype of the virus affecting pregnant women, it is recommended that homogeneous cohort or multicenter studies be conducted to generate better evidence regarding the role of dengue as a risk factor for pathological outcomes.”

**R:** It was edited.
